# Return to duty/play after exertional heat injury: do we have all the answers? A lesson from two case studies

**DOI:** 10.1186/s40696-015-0010-3

**Published:** 2015-12-01

**Authors:** Itay Ketko, Amit Druyan, Ran Yanovich, Yoram Epstein, Yuval Heled

**Affiliations:** 1 The Warrior Health Research Institute, Israel Defense Forces, Medical Corps, Ramat Gan, Israel; 2 Heller Institute of Medical Research, Sheba Medical Center, Tel Hashomer, Ramat Gan, Israel; 3 Sackler Faculty of Medicine, Tel Aviv University, Tel Aviv, Israel

**Keywords:** Heat injury, Thermoregulation, Exercise, Heat tolerance test

## Abstract

**Background:**

The common practice in the Israel defense Forces is that exertional heat related injury patients undergo a heat tolerance test 6–8 weeks post event as part of the “return to duty” process. In the case of a positive heat tolerance test the individual is classified as heat intolerant, in some cases however, the thermoregulatory recovery may be longer (several months), and therefore a second heat tolerance test is scheduled 6-8 weeks later. The presented case reports emphasize the possibility of different recovery periods of the thermoregulatory center and the distinction between congenital and acquired physiological heat intolerance.

**Case description:**

Two young healthy males (A and B) were diagnosed with exertional heat related injury during a pre-recruitment sorting process. Both underwent a heat tolerance test, and were found heat intolerant. During the next months they repeated the test several times. Patient A was finally diagnosed as heat tolerant and patient B was diagnosed as heat intolerant.

**Conclusion:**

Susceptibility to heat is a significant determinant for active young people such as athletes and soldiers. Both cases emphasize the importance of the heat tolerance test (and repeated test when needed) as a criteria for an exertional heat related injury patient to return to duty/play and to perform intense physical activities. These cases also emphasize the effectiveness and sensitivity of the test in identifying a temporary and a permanent state of heat intolerance.

## Background

Exertional heat related injuries (EHIs) are a spectrum of clinical disorders that result from accumulation of excess metabolic heat that cannot be dissipated from the body to the environment. This includes heat exhaustion and exertional heat stroke (EHS) [1]. These conditions are often seen among soldiers, athletes and laborers who work under environmental heat stress. It should be emphasized, however, that under certain conditions of exercise and heat stress, even healthy, well acclimated and physically fit individuals will store heat at a rate that will cause a dangerous rise in the rectal temperature (T_rec_) [2–5].

In most cases, EHS patients fully recover without any thermoregulatory sequelae. Some patients, however, even after a complete clinical recovery, are susceptible to heat. This condition is characterized by lower thermoregulatory efficiency and inability to properly adapt to exercising in hot environment. These individuals are defined as heat intolerant (HI) [6, 7]. The phenomenon of heat intolerance is not completely understood and it is suggested to result from either a predisposing inherent impairment of the thermoregulatory mechanisms or as a direct result of the heat stroke itself [6, 8, 9].

The common practice in the Israel Defense Forces (IDF) is that as part of the “return to duty” process all EHI patients undergo a heat tolerance test (HTT) 6-8 weeks following the injury [8, 10]. In general, 100 HTT’s are being performed during 1 year, on average (including repeated tests). The HTT protocol consists of a 2 h treadmill exercise (5 km/h; 2 % grade) in a climatic chamber set to 40 °C and 40 % relative humidity [10]. Under these conditions HI is determined when T_rec_ elevates above 38.5 °C, when heart rate (HR) elevates above 150 beats per minute (bpm), or when either do not tend to reach a plateau [11, 12]. Those individuals whose HTT is negative are defined as heat tolerant and can gradually return to duty according to a personally tailored training program. Those who have abnormal positive HTT results are classified as HI and will prohibited from performing any intense activity until taking a second HTT 3–6 months after the first test. If the second HTT is negative the temporary prohibition from any intense activity is aborted and they can gradually return to full duty. However, if the result of the HTT is still positive, then a permanent military medical profile is adjusted to prevent his return to a physically active military combat service.

In some rare cases, depending on the second HTT result, an additional HTT may be scheduled a few months later [13]. Nevertheless, because of the small numbers of patients undergoing more than 2 tests, a follow up study of a large cohort of HI individuals has never been performed and the efficacy of a third HTT has never been evaluated. In this manuscript we describe two cases that emphasize the complexity of the heat intolerance diagnosis and the return to duty/play after EHS.

## Case description

### Case number 1 (candidate A)

A young apparently healthy, physically fit male (19 years old, height: 173 cm; weight: 65 kg), collapsed during a military sorting process. His first T_rec_ measurement, a few minutes after collapse, was 39.5 °C; and due to the accompanied condition, together with the sequence of events, he was diagnosed as EHI. His body temperature was quickly reduced by spraying copious amount of tap water and after a few hours of supervision he was released from the base-clinic but refrained from physical exertions until a HTT was scheduled.

**Fig. 1:**
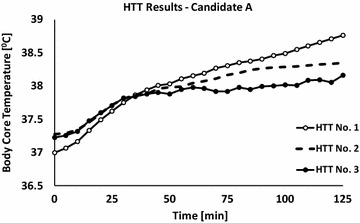
Body core temperature values of candidate ‘*A*’ that were measured during four HTTs. First test: −∘−; Second test: - -; Third test: −•−; data are presented at a sampling rate of 1–5 min

**Fig. 2:**
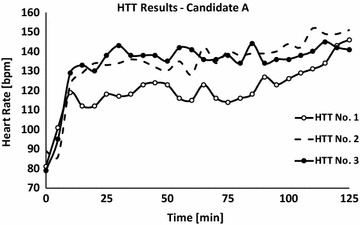
Heart rate values of candidate ‘*A*’ that were measured during four HTTs. First test: −∘−; second test: - -; third test: −•−; data are presented at a sampling rate of 1–5 min

Six weeks after the event he underwent a HTT. During the test, the patient’s T_rec_ did not tend to plateau and the final T_rec_ was above 38.5 °C (Fig. 1). The HR dynamics was similar to that of the T_rec_ (Fig. 2). Accordingly, the patient was diagnosed as temporary HI and a temporary military medical profile was adjusted to prevent him from performing intense physical activity until a second evaluation.

Three months after the first HTT the patient underwent a second test. The test was summarized as positive, mainly because of the endpoint HR (Fig. 2), that was higher than the upper threshold limit for a negative HTT (150 bpm), together with a borderline T_rec_ value of 38.3 °C (Fig. 1). To note the patient did not show any signs of anhidrosis; average sweat rate was 556 ± 35 g/h [314 ± 20 g/(h m^2^)] (average sweat rate during a HTT is 775 ± 194 g/h [411 ± 92 g/(h m^2^)]). Due to the fact that the last test results were borderline and since the subject insisted to be tested again, another HTT was scheduled 3 months later (8 months after the EHI occurrence).

This 3rd HTT was summarized as negative according to the HTT’s criteria (Figs. 1, 2); accordingly, the soldier’s military medical profile was adjusted (post EHS with full recovery followed by a normal HTT) and he was approve to return to duty and gradually participate in physical demanding exercises according to a personally adjusted training program.

### Case number 2 (candidate B)

A young (20 years old, height: 186 cm; weight: 90 kg), apparently healthy, averagely fit male (he trained frequently during the year prior to the event and lost 20 kg of his body weight). He collapsed during a vigorous exercise as part of a military pre-recruitment sorting process. His T_rec_ upon collapse was 40 °C and he was diagnosed as EHS. The patient was immediately cooled by spraying copious amount of tap water. When Trec of 37 °C was measured he was evacuated to a near hospital. An investigation of the case revealed sleep deprivation and apparently mild hypohydration.

**Fig. 3:**
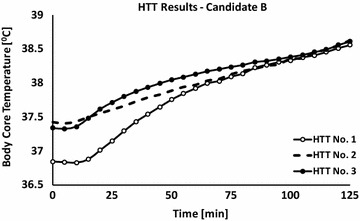
Body core temperature values of candidate ‘*B*’ that were measured during three HTTs. First test: −∘−; second test: - -; third test: −•−; data are presented at a sampling rate of 1–5 min

**Fig. 4:**
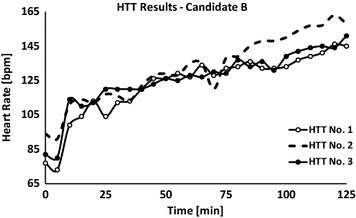
Heart rate values of candidate ‘*B*’ that were measured during the three HTTs. First test: −∘−; second test: - -; third test: −•−; data are presented at a sampling rate of 1–5 min

Six weeks after the event, an HTT was performed. The results indicated HI (Figs. 3, 4) and during the next 10 months he repeated the test twice. Those HTTs were also positive and the patient was finally classified as HI. To note, the patient’s sweat rate (averaging all three HTTs) was within the normal expected range [695 ± 53 g/h (324 ± 24 g/(h m^2^)]. His military medical profile was adjusted to accommodate this condition, which prevented him from intense physically activities during military service.

## Discussion

The underlying reasons for HI are yet not fully understood. The decision to allow the individual to return to training and engage in intense physical activities should be determined, under predetermined conditions, by valid physiological criteria (mostly T_rec_ and HR) that define one’s tolerance to perform exercise under heat load conditions. Presently, the IDF’s HTT protocol provides the only validated scientific tool to diagnose HI. This test has been adopted along the years by other institutes around the world and is being used as a clinically diagnostic tool for back to duty decisions [2, 14]. Nevertheless, when associated with prior EHI the recovery period may vary.

The case reports demonstrate the need to properly return an EHI patient to duty/play and to consider further follow-up before prohibiting someone from intense physical exercise.

Both cases were presented as HI 6–8 weeks after the event. For subject A, the thermoregulatory mechanisms have not fully recovered even after four and a half months after the collapse. However, a certain recovery expressed by a decrease of T_rec_ values can be seen along the tests compared to the first HTT. Only 8 months after collapsing, his HTT results showed a physiological response within the normal range indicative to a physiological recovery. It should be noted, however, that while T_rec_ values during the last three HTTs were lower compared to the first HTT, HR values were higher. This may result from increased reactivity of the cardiovascular system, a key component in regulating T_rec_ or due to a decline in physical fitness.

The case of subjects A emphasizes the importance of performing at least two tests before a final diagnosis of HI can be made. We suggest that any case with borderline results or if a tendency of improvement is manifested in the second HTT, a third test should be considered (6–9 months after the injury). This period may allow sufficient time for physiological recovery.

When comparing subject A first and last HTT (six and a half months between the tests), it can be concluded that HI was probably not congenital but associated with the heat injury. On the other hand, in the case of subject B, heat intolerance was observed in subsequent HTTs even 11 months after the EHI. What seems like permanent heat intolerance could have been either a sequel of the EHS or a congenital deficiency in the thermoregulatory mechanisms. The latter can explain the EHS during the exposure to exercise under heat load conditions. Moreover, it might be speculated that the severity of the EHI (higher T_rec_ value in subject B compared to subject A, at the time of collapse) is associated with longer recovery time of the thermoregulatory mechanism.

## Conclusions

The two cases presented in this manuscript demonstrate that the HTT is an effective and sensitive screening tool for identifying both temporary and permanent states of HI and emphasize the importance and relevance of performing a HTT (and repeated tests when needed) after EHI, in order to determine the state of tolerance to heat before returning to duty/play.

Nevertheless, the ability neither to predict recovery time after EHS nor to understand the mechanisms that cause HI is still unattainable. At this point, encouraging performing an HTT after heat injuries and in case of positive results to repeat the test every 3 months for at least 1 year is the recommended policy.

Due to the unfamiliarity of this complex phenomenon among physicians, both military and civilian, and due to the increasing number of participants (and heat injuries) in extreme sport events in the general population, we suggest that this issue will be further discussed and considered by clinicians when they are required to have a decision. We also point to the necessity to find biological/molecular biomarkers that will assist in evaluating the recovery after EHS. These markers may also further contribute to a better understanding of HI mechanism and physiological recovery process following heat injuries.

## Consent

Written informed consent was obtained from the patient for publication of this case report and any accompanying images. A copy of the written consent is available for review by the Editor-in-Chief of this journal.

## Abbreviations

EHI: exertional heat injury
EHS: exertional heat stroke
T_rec_: rectal temperature
HI: heat intolerant/intolerance
IDF: Israel Defense Force
HTT: heat tolerance test
HR: heart rate
Bpm: beats per minute
